# IgA class-switched CD27^−^CD21^+^ B cells in IgA nephropathy

**DOI:** 10.1093/ndt/gfae173

**Published:** 2024-07-17

**Authors:** Anna Popova, Baiba Slisere, Karlis Racenis, Viktorija Kuzema, Roberts Karklins, Mikus Saulite, Janis Seilis, Anna Jana Saulite, Aiga Vasilvolfa, Kristine Vaivode, Dace Pjanova, Juta Kroica, Harijs Cernevskis, Aivars Lejnieks, Aivars Petersons, Kristine Oleinika

**Affiliations:** Department of Nephrology, Pauls Stradins Clinical University Hospital, Riga, Latvia; Department of Biology and Microbiology, Riga Stradins University, Riga, Latvia; Department of Internal Medicine, University of Latvia, Riga, Latvia; Joint Laboratory, Pauls Stradins Clinical University Hospital, Riga, Latvia; Department of Doctoral Studies, Riga Stradins University, Riga, Latvia; Department of Nephrology, Pauls Stradins Clinical University Hospital, Riga, Latvia; Department of Biology and Microbiology, Riga Stradins University, Riga, Latvia; Department of Internal Diseases, Riga Stradins University, Riga, Latvia; Department of Nephrology, Pauls Stradins Clinical University Hospital, Riga, Latvia; Department of Internal Diseases, Riga Stradins University, Riga, Latvia; Department of Internal Diseases, Riga Stradins University, Riga, Latvia; Department of Nephrology, Pauls Stradins Clinical University Hospital, Riga, Latvia; Department of Internal Diseases, Riga Stradins University, Riga, Latvia; Department of Nephrology, Pauls Stradins Clinical University Hospital, Riga, Latvia; Department of Nephrology, Pauls Stradins Clinical University Hospital, Riga, Latvia; Department of Nephrology, Pauls Stradins Clinical University Hospital, Riga, Latvia; Department of Internal Medicine, University of Latvia, Riga, Latvia; Department of Internal Diseases, Riga Stradins University, Riga, Latvia; Institute of Microbiology and Virology, Riga Stradins University, Riga, Latvia; Institute of Microbiology and Virology, Riga Stradins University, Riga, Latvia; Department of Biology and Microbiology, Riga Stradins University, Riga, Latvia; Department of Nephrology, Pauls Stradins Clinical University Hospital, Riga, Latvia; Department of Internal Diseases, Riga Stradins University, Riga, Latvia; Department of Internal Diseases, Riga Stradins University, Riga, Latvia; Riga East Clinical University Hospital, Riga, Latvia; Department of Nephrology, Pauls Stradins Clinical University Hospital, Riga, Latvia; Department of Internal Diseases, Riga Stradins University, Riga, Latvia; Department of Internal Diseases, Riga Stradins University, Riga, Latvia; Program in Cellular and Molecular Medicine, Boston Children's Hospital, Harvard Medical, School, Boston, MA, USA

**Keywords:** antibodies, B cells, disease mechanisms, IgA nephropathy, IgA-producing plasmablasts

## Abstract

**Background:**

Immunoglobulin A nephropathy (IgAN) is characterized by the production of galactose-deficient IgA1 (GdIgA1) antibodies. As the source of pathogenic antibodies, B cells are central to IgAN pathogenesis, but the B cell activation pathways as well as the potential B cell source of dysregulated IgA secretion remain unknown.

**Methods:**

We carried out flow cytometry analysis of peripheral blood B cells in patients with IgAN and control subjects with a focus on IgA-expressing B cells to uncover the pathways of B cell activation in IgAN and how these could give rise to pathogenic GdIgA1 antibodies.

**Results:**

In addition to global changes in the B cell landscape—expansion of naïve and reduction in memory B cells—IgAN patients present with an increased frequency of IgA-expressing B cells that lack the classical memory marker CD27, but are CD21^+^. IgAN patients furthermore have an expanded population of IgA^+^ antibody-secreting cells, which correlate with serum IgA levels. Both IgA^+^ plasmabalsts and CD27^−^ B cells co-express GdIgA1. Implicating dysregulation at mucosal surfaces as the driver of such B cell differentiation, we found a correlation between lipopolysaccharide in the serum and IgA^+^CD27^−^ B cell frequency.

**Conclusion:**

We propose that dysregulated immunity in the mucosa may drive *de novo* B cell activation within germinal centres, giving rise to IgA^+^CD27^−^ B cells and subsequently IgA-producing plasmablasts. These data integrate B cells into the paradigm of IgAN pathogenesis and allow further investigation of this pathway to uncover biomarkers and develop therapeutic interventions.

## Graphical Abstract

**Graphical Abstract:**
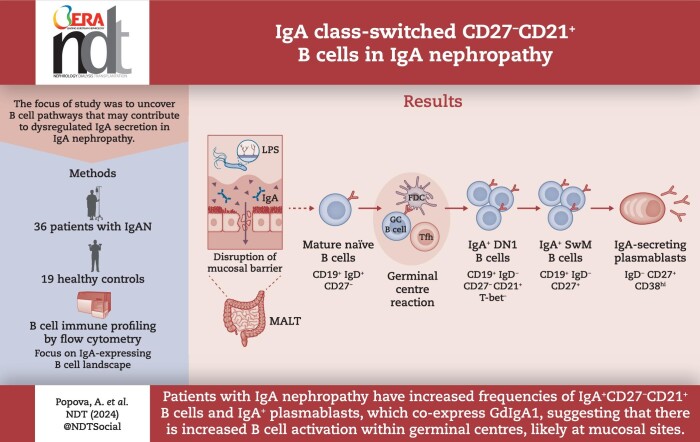

KEY LEARNING POINTS **What was known:**

- Patients with immunoglobulin A nephropathy (IgAN) have aberrant production of galactose-deficient IgA1 (GdIgA1) and antibodies against it, which together form immune complexes that are deposited in the renal mesangium and lead to kidney damage; this is known as the multi-hit model of IgAN pathogenesis.
- The multi-hit model centrally implicates B cells as they produce both GdIgA1 and antibodies against it, yet B cell activation pathways that lead to aberrant antibody production are absent from the model.
- Only isolated reports exist describing specific features of B cells that are altered in patients with IgAN, including a reduction in regulatory B cells, increase in toll-like receptor 7 expression in total peripheral blood B cells and elevated frequency of circulating CCR9^+^IgA^+^ B cells.

**This study adds:**

- In addition to changes in the overall circulating B cell landscape, differentiation of IgA^+^ plasmablasts is enhanced in patients with IgAN and their levels correlate with serum IgA.
- IgA-expressing plasmablast frequency correlates with that of IgA^+^CD21^+^ B cells, which lack the classical memory B cell marker CD27.
- Both IgA^+^ plasmablasts and IgA-expressing CD27^−^ B cells co-express GdIgA1.
- IgA^+^CD27^−^CD21^+^ B cell frequency correlates with serum lipopolysaccharide levels, implicating mucosa in their activation.

**Potential impact:**

- We uncover the previously unknown B cell activation pathway that appears to be associated with pathogenic IgA secretion in IgAN and integrate this into the multi-hit model of IgAN pathogenesis.
- This pathway holds potential for further investigation to identify biomarkers and therapeutic targets in IgAN.

## INTRODUCTION

Immunoglobulin A nephropathy (IgAN) is an autoimmune disease and the most common form of primary glomerulonephritis with an estimated global incidence of at least 2.5 cases per 100 000 adults annually [1]. Patients present with heterogeneous clinicopathological manifestations and variable prognosis, from asymptomatic changes in urinalysis to rapidly progressive glomerulonephritis [2]. Up to 39% of patients progress to end-stage renal disease (ESRD) over 20 years of follow-up [3]. Present treatments carry severe side-effects and largely fail to halt the progression of renal decline [4]. The mainstay of therapy is optimized supportive care, i.e. measures that lower blood pressure, reduce proteinuria, minimize lifestyle risk factors and otherwise help to reduce non-specific insults to the kidneys. The use of immunosuppression has become controversial because of its low effectiveness and high rate of serious side effects mainly associated with glucocorticoid treatment [5]. However new treatment regimens with low-dose steroids and enteral targeted-release budesonide for high-risk patients may decrease the rate of IgAN progression [6]. Treatment advances in IgAN have been limited at least in part due to the incomplete understanding of disease aetiopathogenesis.

The multi-hit model of IgAN aetiopathogenesis proposes that IgAN is initiated with the overproduction of galactose-deficient IgA1 (GdIgA1), followed by the development of antibodies against it [7–10]. Together these form immune complexes that lead to nephrotoxicity through complement activation via alternative and lectin pathways. B cells are centrally implicated in the pathogenesis of IgAN as the source of hypoglycosylated IgA1 and autoantibodies against it [11, 12]. Nevertheless, there are crucial gaps in the knowledge on B cell activation and differentiation pathways that lead to secretion of pathogenic IgA antibodies in IgAN. This is particularly striking when compared with the comprehensive understanding gained in other immune-mediated kidney diseases, particularly systemic lupus erythematosus (SLE) [13–15].

Isolated reports have characterized distinct aspects of B cell activation in patients with IgAN. Among the reported B cell alterations have been the reduction in regulatory B cells [16], elevated toll-like receptor 7 (TLR7) expression in circulating B cells [17] and increased frequency of CCR9^+^IgA^+^ B cells in blood [18]. However, B cell activation pathways have not been investigated and remain to be integrated into the paradigm of IgAN aetiopathogenesis. Here we carry out peripheral B cell profiling in a biopsy-confirmed cohort of IgAN patients and healthy controls (HC) to uncover B cell activation pathways that operate in IgAN as well as to determine the cellular pathways implicated in the secretion of pathogenic IgA.

## MATERIALS AND METHODS

### Study participants

Adults with biopsy-confirmed IgAN were recruited at the Nephrology Centre at Pauls Stradins Clinical University Hospital, Riga, Latvia between January 2020 and June 2022. This is the only centre for kidney diseases in Latvia, where IgAN is diagnosed in adults, therefore the IgAN patient cohort is representative of all Latvian adults with IgAN. Age- and sex-matched healthy volunteers were also enrolled. Healthy controls had age-appropriate kidney function, without active urine sediment and proteinuria. Individuals with diabetes mellitus, current pregnancy, severe organ dysfunction, acute cardiovascular disease, hepatic diseases, acute or chronic inflammatory, autoimmune or infectious diseases, immunodeficiency, malignancies, substance and alcohol abuse, kidney replacement therapy (including kidney transplantation) were excluded from the study. All participants provided written informed consent. This study was approved by the Clinical Research Ethics Committee of Pauls Stradins Clinical University Hospital (No 191219-6L) and was performed under the guidance of the Declaration of Helsinki.

### Clinical and laboratory characterization

Serum creatinine, albumin and total cholesterol were measured on Atellica CH (Siemens Healthineers, Erlangen, Germany). Estimated glomerular filtration rate (eGFR) was calculated using the Chronic Kidney Disease Epidemiology Collaboration Creatinine Equation (2021). Serum IgA was measured on Atellica NEPH 630 (Siemens Healthineers, Erlangen, Germany). Proteinuria was determined by spot protein-to-creatinine ratio. Assessment of protein in urine was performed on Cobas Integra 400 Plus (Roche Diagnostics GmbH, Mannheim, Germany). Red blood cell count in urine was determined on Atellica 1500 automated urinalysis system (Siemens Healthineers, Erlangen, Germany). The complete blood counts were performed on ethylenediaminetetraacetic acid (EDTA)-treated peripheral blood samples using UniCel DxH cellular analysis system (Beckman Coulter, Miami, FL, USA). Serum lipopolysaccharide (LPS) levels were detected by enzyme-linked immunosorbent assay (ELISA) (MBS702450 MyBioSource, San Diego, CA, USA) according to the manufacturer's instructions. GdIgA1 levels in serum were measured using an ELISA kit (GdIgA1 Assay Kit-IBL 30111694, IBL International GmBH, Hamburg, Germany) following the manufacturer's instructions. The samples were diluted 200-fold using the provided EIA buffer to obtain biomarker levels within the measurement range of the kit (1.56–100 ng/mL). Blood pressure was measured by a physician during study recruitment.

### Peripheral blood mononuclear cell isolation and serum collection

Peripheral blood was obtained from study participants upon informed consent. Serum from tubes with coagulation activator was isolated by centrifugation and was either used immediately or stored at –80°C. Peripheral blood mononuclear cells (PBMCs) were isolated from heparinized blood by density gradient centrifugation using Histopaque-1077 (Sigma-Aldrich, St Louis, MO, USA). After washing with complete RPMI-1640 (10% fetal bovine serum and 1% penicillin–streptomycin in RPMI-1640), PBMCs were resuspended in freezing media (90% fetal bovine serum and 10% dimethyl sulfoxide) and cryopreserved. All laboratory analyses were done in the Joint Laboratory at Pauls Stradins Clinical University Hospital, Riga, Latvia.

### Immunophenotyping of peripheral blood B cells by flow cytometry

Briefly, for PBMC viability LIVE/DEAD Fixable Near-IR Dead Cell Stain Kit was used (Invitrogen, Waltham, MA, USA). Nonspecific staining was prevented with Fc receptor blocking reagent (Miltenyi Biotec, Bergisch Gladbach, Germany). The cells were incubated with antibodies (details see Supplementary data, Table S1) for 40 min at 4°C; afterwards unbound antibodies were removed by two washes with flow cytometry staining buffer [2% fetal bovine serum and 2 mM EDTA in phosphate-buffered saline (PBS)] and fixed with PBS containing 2% formaldehyde. For intracellular and transcription factor staining, cells were fixed and permeabilized using the Foxp3/Transcription Factor Staining Buffer Set (00-5523-00, Invitrogen, Waltham, MA, USA) followed by incubation with antibodies diluted in permeabilization buffer for 50 min at 4°C. Samples were acquired on the Navios EX flow cytometer (Beckman Coulter, Inc., Brea, CA, USA) and analysed with FlowJo software (BD Life Sciences, Franklin Lakes, NJ, USA). For the detection of GdIgA1 in B cells, we stained PBMCs with AlexaFluor 488-labelled (ReadyLabel Antibody Labeling Kit R10712, Invitrogen, Waltham, MA, USA) monoclonal antibody [GdIgA1 (KM55)-IBL 30117066, IBL Japan, Gunma, Japan] or isotype control (MAB0061, R&D Systems, Minneapolis, MN, USA) for 40 min at 37°C. To evaluate GdIgA1 expression in plasmablasts, cells were permeabilized using the Foxp3/Transcription Factor Staining Buffer Set and stained intracellularly with anti-GdIgA1 antibody or isotype control. Both anti-GdIgA1 and isotype control were used at a final concentration of 67 µg/mL.

### Statistical analysis

All the statistical analyses were conducted using Prism 9 (GraphPad, La Jolla, CA, USA). Data distribution was assessed by the Shapiro–Wilk test and normal Q-Q plots. For normally distributed and homogenous data, independent samples *t*-test was used; when data were not normally distributed, Mann–Whitney U test was used. Fisher's exact test was used to compare sex between groups. Spearman's rank correlation test was used to interrogate statistical significance in correlations. Results were considered statistically significant at *P* < .05.

## RESULTS

To uncover B cell activation pathways and how peripheral B cell composition may be impacted in patients with IgAN, we recruited 36 patients with IgAN and 19 HCs. See Materials and methods for full participant inclusion and exclusion criteria. The demographic, clinical and laboratory data of the cohort are summarized in Table 1. Study participants were sex- and age-matched. Both groups had normal and comparable leukocyte and lymphocyte counts. As expected, IgAN patients had higher serum creatinine levels and lower eGFR than HCs. IgAN patients represented all four chronic kidney disease stages (from 1 to 4) based on eGFR. The median proteinuria of patients with IgAN was 0.48 g/g (interquartile range 0.26–1.35), 11 patients had moderate proteinuria (1–3 g) and only one patient had nephrotic-range proteinuria (>3 g). According to the Oxford classification of IgAN [19], a frequent histological finding was secondary glomerulosclerosis (69.4%). Only in rare cases were tubular atrophy or crescents in <25% of glomeruli seen. Of note, body mass index (BMI) is associated with worse presentation and long-term outcome of IgAN [20, 21], and B cell activation pathways are dysregulated in obesity [22]. In our cohort there were no significant differences in BMI between IgAN patients and controls.

**Table 1:** Demographic, clinical and laboratory characteristics of the study cohort.

Baseline characteristics	HCs, *n* = 19	IgAN patients, *n* = 36	*P*-value
Age, years	49 (23–66, IQR 38–53)	44.5 (22–65, IQR 37–50)	.906
Male, *n* (%)	13 (68.4)	23 (63.9)	.775
BMI, kg/m^2^	26.8 (17.82–40.63, IQR 23.4–31.1)	25.5 (17.6–44.1, IQR 23.4–29.4)	.816
Systolic BP, mmHg	122 (98–145, IQR 120–129)	139 (120–174, IQR 130–152)	<.001
Diastolic BP, mmHg	80 (60–98, IQR 74–85)	85 (70–110, IQR 80–95)	.024
Serum creatinine, µmol/L	83 (55–102, IQR 74–87)	126.5 (49–402, IQR 95,5–214)	<.001
eGFR, mL/min/1.73 m^2^	100 (70–128, IQR 96–107)	56 (15–131, IQR 25.5–85.5)	<.001
CKD stage 1, *n* (%)		6 (16.7)	
CKD stage 2, *n* (%)		12 (33.3)	
CKD stage 3, *n* (%)		7 (19.4)	
CKD stage 4, *n* (%)		11 (30.6)	
Proteinuria, g/g		0.48 (0.07–6.18, IQR 0.26–1.35)	
Haematuria, RBC/µL		21.17 (0–1421, IQR 10–65)	
Serum albumin, g/L		46 (29–51, IQR 42.5–48)	
Serum total cholesterol, mmol/L		5.46 (3.79–8.11, IQR 4.94–5.9)	
Absolute leukocyte count, 10^9^/L	5.3 (4.1–8.4, IQR 4.4–6.5)	7.15 (4.2–12.8, IQR 5.8–7.5)	.07
Absolute lymphocyte count, 10^9^/L	1.9 (1.4–3.0, IQR 1.8–2.4)	1.8 (0.9–3.1, IQR 1.5–2.2)	.291
Oxford classification, *n* (%)			
M1		29 (80.6)	
E1		1 (2.8)	
S1		25 (69.4)	
T1		2 (5.6)	
T2		2 (5.6)	
C1		2 (5.6)	

Data are presented as median, minimum–maximum, interquartile range (IQR), or as *n* (%).

BP: blood pressure; CKD: chronic kidney disease; RBC: red blood cells.

**Figure 1:**
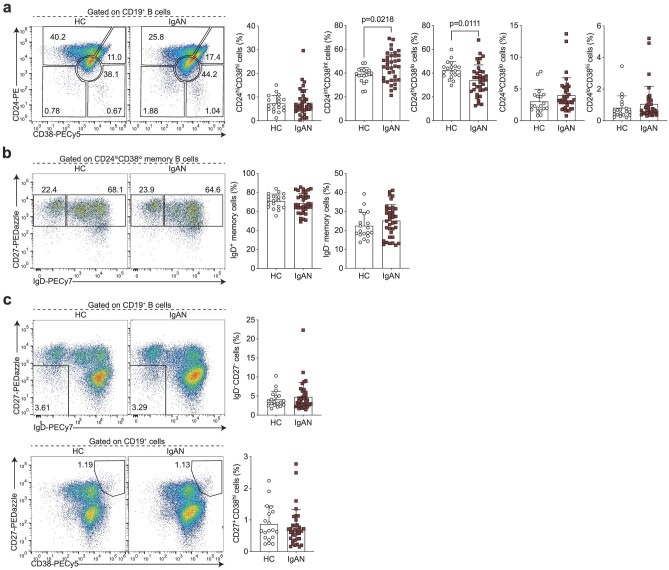
IgAN-associated changes in the peripheral B cell landscape. Representative flow cytometry plots and summary bar charts demonstrating (**a**) the frequencies of transitional (CD24^hi^CD38^hi^), mature (CD24^int^CD38^int^), memory (CD24^hi^CD38^lo^) and activated B cells (CD24^lo^CD38^lo^), and pre-plasmablasts (CD24^lo^CD38^hi^), (**b**) the distribution of memory B cells into IgD^+^ unswitched and IgD^−^ class-switched subsets, (**c**) the frequencies of total double negative (DN; IgD^−^CD27^−^) B cells and plasmablasts (CD27^+^CD38^hi^) in IgAN patients and HCs. Data are mean ± standard deviation and each circle/square represents a study participant. For normally distributed CD24^int^CD38^int^, CD24^hi^CD38^lo^, IgD^+^ memory and IgD^−^ memory B cell populations independent samples *t*-test was used to compare IgAN patients and HCs. For non-normally distributed CD24^hi^CD38^hi^, CD24^lo^CD38^lo^, CD24^lo^CD38^hi^, IgD^−^CD27^−^ and CD27^+^CD38^hi^ B cell subsets, Mann–Whitney U test was used for the comparison of IgAN patients and HCs.

We first carried out B cell phenotyping based on CD24, CD27, CD38 and IgD surface expression. This allowed us to enumerate transitional (CD24^hi^CD38^hi^), mature naïve (CD24^int^CD38^int^), activated (CD24^lo^CD38^lo^) and total memory (CD24^hi^CD38^lo^) B cells, including class-switched (IgD^−^) and unswitched (IgD^+^) subsets, DN (IgD^−^CD27^−^) B cells, pre-plasmablasts (CD24^lo^CD38^hi^) and plasmablasts (CD27^+^CD38^hi^). The CD24/CD38 gating strategy confirmation is shown in Supplementary data, Fig. S1a and b. We found that IgAN patients had a significant increase in mature naïve B cells with a reciprocal decrease in the frequency of total memory B cells (Fig. 1a). Frequencies of switched and unswitched memory B cells were comparable (Fig. 1b). A novel population of IgD^−^CD27^−^ B cells termed DN or atypical memory B cells has been recently described. These DN B cells are expanded in autoimmune conditions, such as SLE, and especially in patients with nephritis [14, 15]. These cells have been shown to be the precursors of autoantibody-producing plasmablasts in SLE. Nevertheless, total DN B cells and plasmablasts were comparable in patients with IgAN and HCs (Fig. 1c).

**Figure 2:**
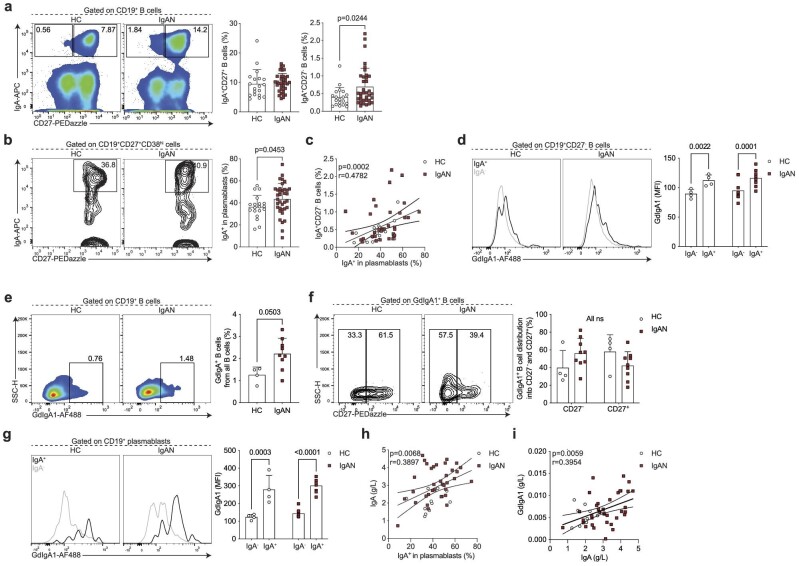
Enhanced differentiation of IgA^+^CD27^−^ B cells and IgA^+^ plasmablasts in IgAN. Representative flow cytometry plots and summary bar charts demonstrating the frequencies of (**a**) IgA^+^CD27^+^ and IgA^+^CD27^−^ B cells, and (**b**) IgA^+^ plasmablasts, (**c**) linear regression analysis of IgA^+^CD27^−^ B cells versus IgA^+^ plasmablasts, and (**d**) representative flow cytometry histograms and summary bar charts demonstrating the median fluorescence intensity of GdIgA1 in IgA^+^ and IgA^−^ CD19^+^CD27^−^ B cells. (**e, f**) Representative flow cytometry plots and summary bar charts demonstrating the frequencies of (**e**) GdIgA1^+^ B cells and (**f**) the distribution of GdIgA1^+^ B cells into CD27^−^ and CD27^+^ subsets. (**g**) Representative flow cytometry histograms and summary bar charts demonstrating the median fluorescence intensity of GdIgA1 in IgA^+^ and IgA^−^ plasmablasts. (h, i) Linear regression analysis of (**h**) serum IgA levels versus IgA^+^ plasmablasts and (**i**) serum GdIgA1 versus serum IgA levels in IgAN patients and HCs. Data are mean ± standard deviation and each circle/square represents a study participant. For non-normally distributed subsets Mann–Whitney U test was used for the comparison of IgAN patients and HCs. Spearman's rank correlation tests were used to interrogate statistical significance in the correlation between IgA^+^CD27^−^ B cells and IgA^+^ plasmablasts, serum IgA levels and IgA^+^ plasmablasts, and serum GdIgA1 and serum IgA levels in IgAN patients and HCs.

The cellular origin and pathway that gives rise to IgA-producing B cells in IgAN is unknown, therefore we next wanted to assay specifically IgA-expressing B cells and antibody-secreting cells (ASCs). That is, we wanted to know if systemic perturbations in the activation and differentiation of IgA-expressing B cells can be detected in patients with IgAN. Among B cells, IgA-expressing classical memory (CD27^+^) B cell frequency was comparable between IgAN patients and HCs (Fig. 2a). However, we noted that in addition to CD27-expressing B cells, there was a smaller population of IgA class-switched B cells that lacked CD27 expression, which was particularly pronounced in IgAN patients. Indeed, there was a significant expansion of these IgA-expressing CD27^−^ B cells in patients with IgAN (Fig. 2a). We also detected a significantly higher frequency of IgA class-switched ASCs in IgAN patients compared with controls (Fig. 2b). Supporting a lineage relationship between IgA-expressing plasmablasts and IgA-expressing CD27^−^ B cells, we found a correlation between these two subsets (Fig. 2c).

We next wanted to address whether the IgA-expressing B cells were indeed expressing GdIgA1. We found that IgA^+^CD27^−^ B cells co-expressed GdIgA1 (Fig. 2d). Reciprocally, in IgAN patients the majority of all GdIgA1^+^ B cells were CD27^−^ (Fig. 2e and f). IgA^+^ plasmablasts also expressed high levels of GdIgA1 (Fig. 2g, Supplementary data, Fig. S1c and d). Finally, implicating the IgA-expressing plasmablast as a potential functional contributor to pathogenesis, we observed a correlation between IgA^+^ plasmablasts and circulating IgA levels (Fig. 2h). Nevertheless, despite the correlation between IgA and GdIgA1, we did not find a relationship between IgA^+^ plasmablasts and serum GdIgA1 levels (Fig. 2i).

**Figure 3:**
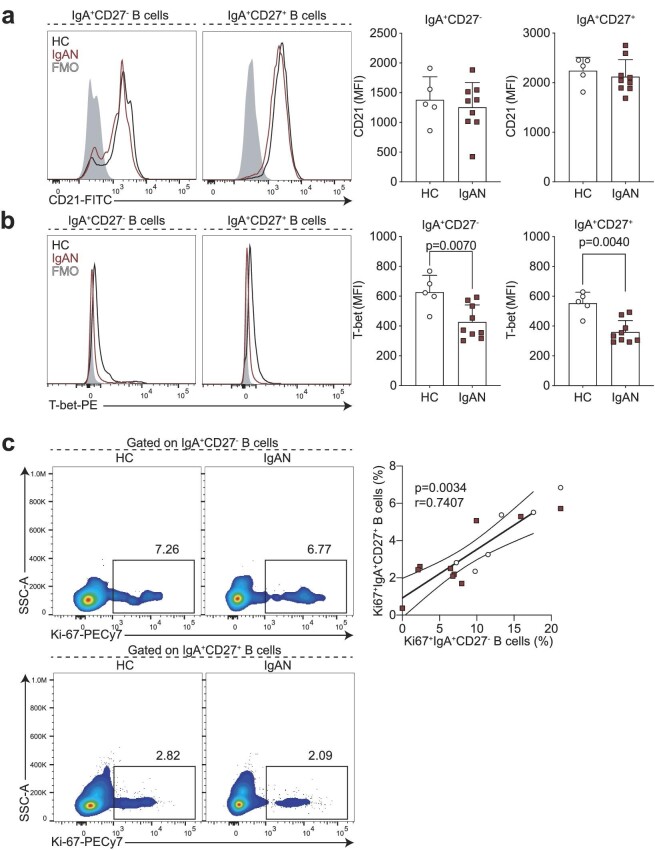
IgA^+^CD27^−^ B cells are phenotypically CD21^+^T-bet^−^. Representative flow cytometry histograms and summary bar charts demonstrating the median fluorescence intensity of CD21 (**a**) and T-bet (**b**) in IgA^+^CD27^−^ and IgA^+^CD27^+^ B cells. (**c**) Representative flow cytometry plots and linear regression analysis of Ki-67^+^IgA^+^CD27^+^ B cells versus Ki-67^+^IgA^+^CD27^−^ B cells in HCs and IgAN patients. Data are mean ± standard deviation and each circle/square represents a study participant. For non-normally distributed CD21 and T-bet mean fluorescence intensity, Mann–Whitney U test was used for the comparison of IgAN patients and HCs. Spearman's rank correlation test was used to interrogate statistical significance in correlations between Ki-67^+^IgA^+^CD27^+^ B cells and Ki-67^+^IgA^+^CD27^−^ B cells in IgAN patients and HCs.

CD27^−^ antigen-experienced B cells comprise two subsets, termed double negative (DN) 1 and 2. DN1 cells are defined by their expression of CD21 and CXCR5, while DN2 B cells lack CD21 and CXCR5 expression and instead express CD11c and are transcriptionally regulated by T-bet [15]. Based on their transcriptional signatures these two subsets of B cells arise from different activation pathways [15]. While DN2 B cells arise through extrafollicular B cell activation, DN1 B cells represent precursors of classical memory B cells that have recently emerged from the germinal centre reaction (and have not yet upregulated CD27). Of note, germinal centres are the microanatomical structures that allow the evolution (by somatic hypermutation of antibody-encoding genes) and selection (affinity maturation) of B cells that enable the production of high-affinity antibodies [23]. The understanding of which pathway B cells are activated through can elucidate factors that regulate the response (e.g. T cell help) or properties of the compartment (e.g. longevity) [23]. We found that the IgA^−^CD27^+^ B cells were phenotypically CD21^hi^ and T-bet^lo^ corresponding to DN1 phenotype (Fig. 3a and b), suggesting they may indeed be the precursors of IgA^+^CD27^+^ classical memory B cells. Of note, we used IgA^+^CD27^+^ memory B cells as a control for high expression of CD21 and lack of T-bet. To further interrogate this developmental relationship, we reasoned that most of the classical memory compartment (CD27^+^) in patients with IgAN and HCs would be composed of foreign antigen–specific B cells generated throughout the lifetime. The majority of this pool should be resting cells, apart from those that report and participate in an ongoing immune response. We then carried out Ki-67 staining and found a significant positive correlation between proliferating IgA^+^ DN1 and Ki-67^+^IgA^+^CD27^+^ memory B cells (Fig. 3c). Therefore, the shared phenotypic and proliferation characteristics of these subsets suggest a developmental relationship and support increased generation of IgA-expressing ASCs through the germinal centre pathway.

**Figure 4:**
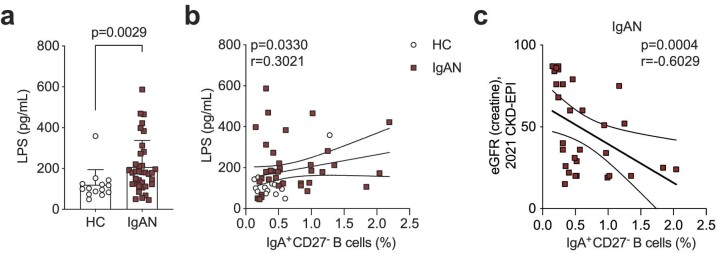
Serum LPS levels correlate with IgA^+^CD27^−^ B cell frequency. (**a**) Summary bar chart showing serum LPS levels and (**b**) linear regression analysis of serum LPS levels versus IgA^+^CD27^−^ B cells in IgAN patients and HCs. (**c**) Linear regression analysis of eGFR versus IgA^+^CD27^−^ B cells in IgAN patients. Data are mean ± standard deviation and each circle/square represents a study participant. For non-normally distributed serum LPS levels Mann–Whitney U test was used for the comparison of IgAN patients and HCs. Spearman's rank correlation test was used to interrogate statistical significance in the correlation between serum LPS levels and IgA^+^CD27^−^ B cells in IgAN patients and HCs.

Finally, we wanted to explore the mucosal–kidney axis in relation to B cell activation in IgAN and ask if previously reported perturbations at mucosal surfaces were linked to this DN1 B cell differentiation pathway. LPS is known not only to influence B cell class-switching to IgA [24, 25] but is also used as a surrogate marker of dysbiosis and gut permeability [26–29]. We found that LPS was significantly elevated in the serum of IgAN patients compared with HCs (Fig. 4a), confirming previously published data [30]. We further found that IgA-expressing CD27^−^ B cells correlated with serum LPS levels (Fig. 4b; the relationships between LPS and the other B cell subsets examined in this study are presented in Supplementary data, Table S2). This suggests LPS may either directly drive their expansion/class-switching or that the observed correlation is because both increased LPS and IgA^+^CD27^−^ B cells are a consequence of mucosal dysbiosis/reduced barrier function, which contributes to IgA-expressing plasmablast differentiation. Finally, we wanted to ask how the expansion of IgA^+^CD27^−^ B cells related to clinical features of IgAN. We found that specifically in patients with reduced kidney function (eGFR <90 mL/min) there was an inverse correlation between eGFR and the frequency of IgA^+^CD27^−^ B cells (Fig. 4c).

## DISCUSSION

**Figure 5:**
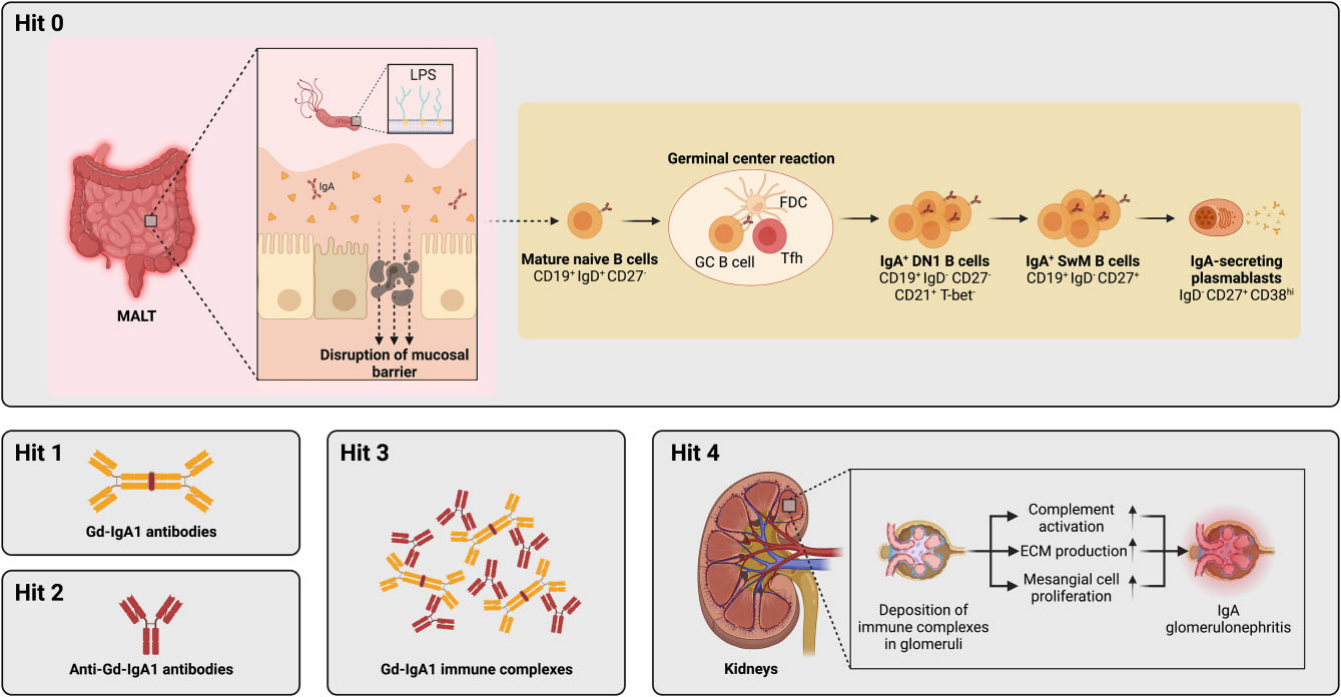
Revised multi-hit model with integration of B cell activation pathways. Hit 1 of the multi-hit pathogenesis is the production of pathogenic GdIgA1. This is followed by production of anti-IgA antibodies (Hit 2) and formation of immune complexes (Hit 3), which are finally deposited in the kidney glomeruli and cause tissue damage and renal decline. Here we have uncovered the B cell pathway that leads to GdIgA1 production (Hit 1) in IgAN pathogenesis, and term it ‘Hit 0’. Specifically, naïve B cells are *de novo* activated within germinal centres and give rise to IgA^+^CD27^−^ DN1 B cells and subsequently IgA-expressing plasmablasts which secrete pathogenic IgA.

The multi-hit model is the blueprint for explaining IgAN aetiopathogenesis [7–10]. Advances have been made particularly in uncovering the mechanisms operating within the kidney that contribute to organ damage (Hit 4, see Fig. 5). These include the dissection of how immune complexes containing IgA bind to mesangial cells, triggering proliferation and increased synthesis of extracellular matrix components as well as the role of the CD89, the IgA Fc receptor, in augmenting tissue damage [31, 32]. However, these mechanistic insights are unlikely to provide the key to therapeutic advances as they operate once B cell tolerance is already broken.

We report here that global alterations in the B cell compartment are present in patients with IgAN, specifically the expansion of naïve B cells and a reduction in total memory B cells. This observation is of significance as IgAN is considered an organ-specific autoimmune disease, yet these data support that systemic immunity is dysregulated beyond that which has been previously described at mucosal sites [33–35]. Increase in naïve B cells and reduction in total memory B cells is characteristic of systemic autoimmune diseases such as SLE and conditions associated with chronic low-grade inflammation, including obesity [15, 22]. However, we did not observe an expansion of total DN B cells linked to more severe systemic autoimmune and inflammatory phenotypes. It will be important to address whether the systemic changes in the overall B cell landscape we report here are causal to IgAN or a consequence of autoimmunity-associated inflammation/bystander effects.

As the source of GdIgA1 antibodies, B cells are a promising target in IgAN, but B cell activation and differentiation pathways that lead to pathogenic IgA production had remained uncharacterized. Uncovering how B cells are affected and become activated is thus important for the complete understanding of IgAN pathogenesis, prognosis and treatment. Here we provide mechanistic insight into the early steps of IgAN aetiopathogenesis by demonstrating that IgA-expressing B cells that lack the classical memory marker CD27 but express CD21 are expanded in patients with IgAN and appear to be the cellular precursors of IgA-producing plasmablasts. We show that IgA^+^CD27^−^CD21^+^ B cells are apparently recent emigrants from ongoing germinal centres, which suggests the continuous output of the pathogenic precursors, rather than dominant contribution by memory B cells and ASCs differentiated at an earlier timepoint. Class-switched CD27^−^CD21^+^ DN1 B cells have not been previously associated with disease states (unlike the extensively characterized DN2 subset), therefore this work may help uncover more about this B cell population. This observation of increased recent germinal centre emigrant DN1 B cells also fits with the report of increased frequency of T follicular helper cells in IgAN patients [36].

Due to their expression of GdIgA1, it is tempting to speculate that IgA^+^CD27^−^ cells indeed participate in disease precipitation, albeit this remains to be experimentally demonstrated. That is, the enhanced generation of IgA^+^CD27^−^ precursors could drive disease or these cells may be generated as part of ongoing inflammation and not causal in IgAN development. Since this study aimed to capture the whole range of IgAN clinical features, our cohort comprised only six patients with eGFR >90 mL/min. We reason that if the B cell dysregulation we characterize here underlies disease, patients would present with an increased activation of this pathway prior to detectable decline in renal function.

That we found a correlation between the CD27^−^ precursors of IgA-expressing plasmablasts and serum LPS levels, implicates mucosal immunity in the differentiation of these cells. LPS could directly contribute to class-switching in activated B cells as previously observed [24, 25]. In support of direct activation, tonsillar B cells from IgAN patients have been shown to produce increased IgA in response to LPS stimulation [37]. Others have shown that upon *in vitro* culture with LPS, B cells downregulate the expression of β3-galactosyltransferase-specific molecular chaperone (Cosmc) [38]. Therefore, LPS may act at multiple levels in B cell activation to not only induce class-switching and IgA production, but to also regulate IgA glycosylation. Another possible explanation for the correlation between IgA^+^CD27^−^ B cells and LPS is that both are increased as a consequence of intestinal inflammatory processes that may result in enhanced generation of IgA^+^CD27^−^ B cells and increased intestinal permeability. Supporting the mucosa as a site of origin for these IgA-expressing B cells, IgAN patients have an increased frequency of IgA^+^ B cells that express the mucosal homing marker CCR9 in peripheral blood [18]. It is possible that tonsillectomy, a procedure that has been demonstrated to be of some benefit in IgAN [39, 40], may eliminate the niche of either generation or homing of these B cells as has been speculated by others [41]. It may also provide clues as to why B cell depletion has shown variable success in IgAN despite the central role of B cells in the disease pathogenesis [42]—the mucosal niches may harbour the majority of precursors that are not efficiently depleted by the B cell targeting therapy. Albeit the literature is not consistent, studies have suggested that rituximab may be less effective in depleting B cells within lymphoid tissues [43]. Additionally, in other autoimmune diseases both memory B cells and B cells *de novo* engaging in immune responses—such as germinal centre B cells which are the proposed progenitors of DN1 B cells—have been shown to be resistant to rituximab treatment [44, 45]. Recent findings of disease-modifying effects of enteral budesonide in IgAN further implicate mucosal sites in driving immunopathogenesis [46]. Whether budesonide affects B cell phenotypes remains to be determined, but it is tempting to speculate that it may target IgA^+^ ASC precursors at mucosal sites.

In IgAN endocapillary proliferation, tubular atrophy/interstitial fibrosis are independent predictors of rate of loss of renal function [47]. In our cohort of IgAN patients, the prevalence of these findings was relatively low. It is important to note that kidney biopsy may have been conducted several years before the patient's visit. Sex distribution, age and BMI in our cohort were similar to those reported by other studies evaluating B cells in IgAN; clinically, patients exhibited lower protein excretion despite reduced eGFR [16, 18]. Of note, a limitation of this study is that our cohort consisted entirely of white Eastern Europeans and thus the B cell activation pathway we have uncovered remains to be investigated in non-European cohorts.

Now that we have identified the aberrant B cell differentiation pathway in IgAN, important further questions can be addressed. With regards to the antigen specificity and clonality of IgA-expressing B cells—what are the B cells recognizing (e.g. food, microbial antigens) and how diverse is the IgA response within an individual and between individuals? With respect to the site of induction and long-term residence—where are the IgA-expressing plasmablast precursors induced and maintained (e.g. mucosa, bone marrow) and is this shared across IgAN patients? How are pathogenic IgA^+^CD27^−^ B cells different from foreign-antigen reactive IgA^+^CD27^+^ B cells—can targets be identified to specifically deplete or inhibit the differentiation of pathogenic B cells?

In summary, we propose that dysregulation of mucosal immunity may drive the increased naïve B cell activation in germinal centres, giving rise to IgA^+^CD27^−^CD21^+^ B cells and subsequently IgA-producing plasmablasts; this pathway can be further explored for biomarkers and therapeutic targets in IgAN.

## Supplementary Material

gfae173_Supplemental_Files

## Data Availability

The data that support the findings of this study are available in the article and in its online Supplementary data.

## References

[1] McGrogan A, Franssen CF, de Vries CS. The incidence of primary glomerulonephritis worldwide: a systematic review of the literature. Nephrol Dial Transplant 2011;26:414–30. 10.1093/ndt/gfq665 21068142

[2] Lafayette RA, Kelepouris E. Immunoglobulin A nephropathy: advances in understanding of pathogenesis and treatment. Am J Nephrol 2018;47 **Suppl**:43–52. 10.1159/000481636 29852501

[3] Berthoux FC, Mohey H, Afiani A. Natural history of primary IgA nephropathy. Semin Nephrol 2008;28:4–9. 10.1016/j.semnephrol.2007.10.001 18222341

[4] Gleeson PJ, O'Shaughnessy MM, Barratt J. IgA nephropathy in adults—treatment standard. Nephrol Dial Transplant 2023;38:2464–73 . 10.1093/ndt/gfad146 37418237 PMC10794095

[5] Ponticelli C, Locatelli F. Corticosteroids in IgA nephropathy. Am J Kidney Dis 2018;71:160–2. 10.1053/j.ajkd.2017.10.004 29203129

[6] Floege J, Rauen T, Tang SCW. Current treatment of IgA nephropathy. Semin Immunopathol 2021;43:717–28. 10.1007/s00281-021-00888-3 34495361 PMC8551131

[7] Suzuki H, Kiryluk K, Novak J et al. The pathophysiology of IgA nephropathy. J Am Soc Nephrol 2011;22:1795–803. 10.1681/ASN.2011050464 21949093 PMC3892742

[8] Magistroni R, D'Agati VD, Appel GB et al. New developments in the genetics, pathogenesis, and therapy of IgA nephropathy. Kidney Int 2015;88:974–89. 10.1038/ki.2015.252 26376134 PMC4653078

[9] Gentile M, Sanchez-Russo L, Riella LV et al. Immune abnormalities in IgA nephropathy. Clin Kidney J 2023;16:1059–70. 10.1093/ckj/sfad025 37398689 PMC10310525

[10] Wyatt RJ, Julian BA. IgA nephropathy. N Engl J Med 2013;368:2402–14. 10.1056/NEJMra1206793 23782179

[11] Novak J, Julian BA, Tomana M et al. IgA glycosylation and IgA immune complexes in the pathogenesis of IgA nephropathy. Semin Nephrol 2008;28:78–87. 10.1016/j.semnephrol.2007.10.009 18222349 PMC2241661

[12] Moldoveanu Z, Wyatt RJ, Lee JY et al. Patients with IgA nephropathy have increased serum galactose-deficient IgA1 levels. Kidney Int 2007;71:1148–54. 10.1038/sj.ki.5002185 17342176

[13] Oleinika K, Mauri C, Salama AD. Effector and regulatory B cells in immune-mediated kidney disease. Nat Rev Nephrol 2019;15:11–26. 10.1038/s41581-018-0074-7 30443016

[14] Tipton CM, Fucile CF, Darce J et al. Diversity, cellular origin and autoreactivity of antibody-secreting cell population expansions in acute systemic lupus erythematosus. Nat Immunol 2015;16:755–65. 10.1038/ni.3175 26006014 PMC4512288

[15] Jenks SA, Cashman KS, Zumaquero E et al. Distinct effector B cells induced by unregulated toll-like receptor 7 contribute to pathogenic responses in systemic lupus erythematosus. Immunity 2018;49:725–39.e6. 10.1016/j.immuni.2018.08.015 30314758 PMC6217820

[16] Wang YY, Zhang L, Zhao PW et al. Functional implications of regulatory B cells in human IgA nephropathy. Scand J Immunol 2014;79:51–60. 10.1111/sji.12128 24219615

[17] Sendic S, Mansouri L, Lundberg S et al. B cell and monocyte phenotyping: a quick asset to investigate the immune status in patients with IgA nephropathy. PLoS One 2021;16:e0248056. 10.1371/journal.pone.0248056 33740017 PMC7978284

[18] Sallustio F, Curci C, Chaoul N et al. High levels of gut-homing immunoglobulin A+ B lymphocytes support the pathogenic role of intestinal mucosal hyperresponsiveness in immunoglobulin A nephropathy patients. Nephrol Dial Transplant 2021;36:452–64. 10.1093/ndt/gfaa264 33200215 PMC7898021

[19] Trimarchi H, Barratt J, Cattran DC et al. Oxford Classification of IgA nephropathy 2016: an update from the IgA Nephropathy Classification Working Group. Kidney Int 2017;91:1014–21. 10.1016/j.kint.2017.02.003 28341274

[20] Kataoka H, Ohara M, Shibui K et al. Overweight and obesity accelerate the progression of IgA nephropathy: prognostic utility of a combination of BMI and histopathological parameters. Clin Exp Nephrol 2012;16:706–12. 10.1007/s10157-012-0613-7 22350469

[21] Hong YA, Min JW, Ha MA et al. The impact of obesity on the severity of clinicopathologic parameters in patients with IgA nephropathy. J Clin Med 2020;9:2824. 10.3390/jcm9092824 32878271 PMC7564413

[22] Slisere B, Arisova M, Aizbalte O et al. Distinct B cell profiles characterise healthy weight and obesity pre- and post-bariatric surgery. Int J Obes 2023;47:970–8. 10.1038/s41366-023-01344-y PMC10511309 37463992

[23] Elsner RA, Shlomchik MJ. Germinal center and extrafollicular B cell responses in vaccination, immunity, and autoimmunity. Immunity 2020;53:1136–50. 10.1016/j.immuni.2020.11.006 33326765 PMC7748291

[24] Stavnezer J, Guikema JE, Schrader CE. Mechanism and regulation of class switch recombination. Annu Rev Immunol 2008;26:261–92. 10.1146/annurev.immunol.26.021607.090248 18370922 PMC2707252

[25] Cerutti A . The regulation of IgA class switching. Nat Rev Immunol 2008;8:421–34. 10.1038/nri2322 18483500 PMC3062538

[26] Scaldaferri F, Lopetuso LR, Petito V et al. Gelatin tannate ameliorates acute colitis in mice by reinforcing mucus layer and modulating gut microbiota composition: emerging role for ‘gut barrier protectors’ in IBD? United European Gastroenterol J 2014;2:113–22. 10.1177/2050640614520867 PMC4040816 24918016

[27] Zhao H, Zhang H, Wu H et al. Protective role of 1,25(OH)2 vitamin D3 in the mucosal injury and epithelial barrier disruption in DSS-induced acute colitis in mice. BMC Gastroenterol 2012;12:57. 10.1186/1471-230X-12-57 22647055 PMC3464614

[28] Yu LC, Flynn AN, Turner JR et al. SGLT-1-mediated glucose uptake protects intestinal epithelial cells against LPS-induced apoptosis and barrier defects: a novel cellular rescue mechanism? FASEB J 2005;19:1822–35. 10.1096/fj.05-4226com 16260652

[29] Nighot M, Al-Sadi R, Guo S et al. Lipopolysaccharide-induced increase in intestinal epithelial tight permeability is mediated by toll-like receptor 4/myeloid differentiation primary response 88 (MyD88) activation of Myosin light chain kinase expression. Am J Pathol 2017;187:2698–710. 10.1016/j.ajpath.2017.08.005 29157665 PMC5718096

[30] Tang Y, Zhu Y, He H et al. Gut dysbiosis and intestinal barrier dysfunction promotes IgA nephropathy by increasing the production of Gd-IgA1. Front Med 2022;9:944027. 10.3389/fmed.2022.944027 PMC9302483 35872757

[31] Launay P, Grossetete B, Arcos-Fajardo M et al. Fcalpha receptor (CD89) mediates the development of immunoglobulin A (IgA) nephropathy (Berger's disease). Evidence for pathogenic soluble receptor-iga complexes in patients and CD89 transgenic mice. J Exp Med 2000;191:1999–2010. 10.1084/jem.191.11.1999 10839814 PMC2213528

[32] Van der Steen LP, Bakema JE, Sesarman A et al. Blocking fcalpha receptor I on granulocytes prevents tissue damage induced by IgA autoantibodies. J Immunol 2012;189:1594–601. 10.4049/jimmunol.1101763 22802416

[33] Bene MC, Hurault De Ligny B, Kessler M et al. Confirmation of tonsillar anomalies in IgA nephropathy: a multicenter study. Nephron 1991;58:425–8. 10.1159/000186474 1922607

[34] Sugiyama N, Shimizu J, Nakamura M et al. Clinicopathological study of the effectiveness of tonsillectomy in IgA nephropathy accompanied by chronic tonsillitis. Acta Otolaryngol 1993;113:43–8. 10.3109/00016489309130265 8285042

[35] Harper SJ, Allen AC, Bene MC et al. Increased dimeric IgA-producing B cells in tonsils in IgA nephropathy determined by in situ hybridization for J chain mRNA. Clin Exp Immunol 2008;101:442–8. 10.1111/j.1365-2249.1995.tb03132.x PMC1553245 7664491

[36] Zhang L, Wang Y, Shi X et al. A higher frequency of CD4(+)CXCR5(+) T follicular helper cells in patients with newly diagnosed IgA nephropathy. Immunol Lett 2014;158:101–8. 10.1016/j.imlet.2013.12.004 24333338

[37] Liu H, Peng Y, Liu F et al. Expression of IgA class switching gene in tonsillar mononuclear cells in patients with IgA nephropathy. Inflamm Res 2011;60:869–78. 10.1007/s00011-011-0347-0 21614556

[38] Qin W, Zhong X, Fan JM et al. External suppression causes the low expression of the Cosmc gene in IgA nephropathy. Nephrol Dial Transplant 2008;23:1608–14. 10.1093/ndt/gfm781 18202089

[39] Hirano K, Matsuzaki K, Yasuda T et al. Association between tonsillectomy and outcomes in patients with immunoglobulin A nephropathy. JAMA Netw Open 2019;2:e194772. 10.1001/jamanetworkopen.2019.4772 31150076 PMC6547111

[40] Feriozzi S, Polci R. The role of tonsillectomy in IgA nephropathy. J Nephrol 2016;29:13–9. 10.1007/s40620-015-0247-4 26582216

[41] Wu G, Peng YM, Liu FY et al. The role of memory B cell in tonsil and peripheral blood in the clinical progression of IgA nephropathy. Hum Immunol 2013;74:708–12. 10.1016/j.humimm.2012.10.028 23313256

[42] Lafayette RA, Canetta PA, Rovin BH et al. A Randomized, controlled trial of rituximab in IgA nephropathy with proteinuria and renal dysfunction. J Am Soc Nephrol 2017;28:1306–13. 10.1681/ASN.2016060640 27821627 PMC5373458

[43] Otten HG, Hans K, Ten M L et al. A single dose of rituximab does not deplete B cells in secondary lymphoid organs but alters phenotype and function. Am J Transplant 2013;13:1503–11. 23570303 10.1111/ajt.12220

[44] Crickx E, Chappert P, Sokal A et al. Rituximab-resistant splenic memory B cells and newly engaged naive B cells fuel relapses in patients with immune thrombocytopenia. Sci Transl Med 2021;13:eabc3961. 10.1126/scitranslmed.abc3961 33853929 PMC7610758

[45] Jiang R, Fichtner ML, Hoehn KB et al. Single-cell repertoire tracing identifies rituximab-resistant B cells during myasthenia gravis relapses. JCI Insight 2020;5:e136471. 10.1172/jci.insight.136471 32573488 PMC7453893

[46] Lafayette R, Kristensen J, Stone A et al. Efficacy and safety of a targeted-release formulation of budesonide in patients with primary IgA nephropathy (NefIgArd): 2-year results from a randomised phase 3 trial. Lancet North Am Ed 2023;402:859–70. 10.1016/S0140-6736(23)01554-4 37591292

[47] Chakera A, MacEwen C, Bellur SS et al. Prognostic value of endocapillary hypercellularity in IgA nephropathy patients with no immunosuppression. J Nephrol 2016;29:367–75. 10.1007/s40620-015-0227-8 26318019

