# Supplementary material for: IgA class-switched CD27−CD21+ B cells in IgA nephropathy
Source: Nephrol Dial Transplant. 2024 Jul 17;40(3):505–15. doi: 10.1093/ndt/gfae173 (PMC11879059; doi:10.1093/ndt/gfae173)
Supplement: gfae173_Supplemental_Files [file gfae173_supplemental_files.zip › Suppl. Figure 1..pdf]

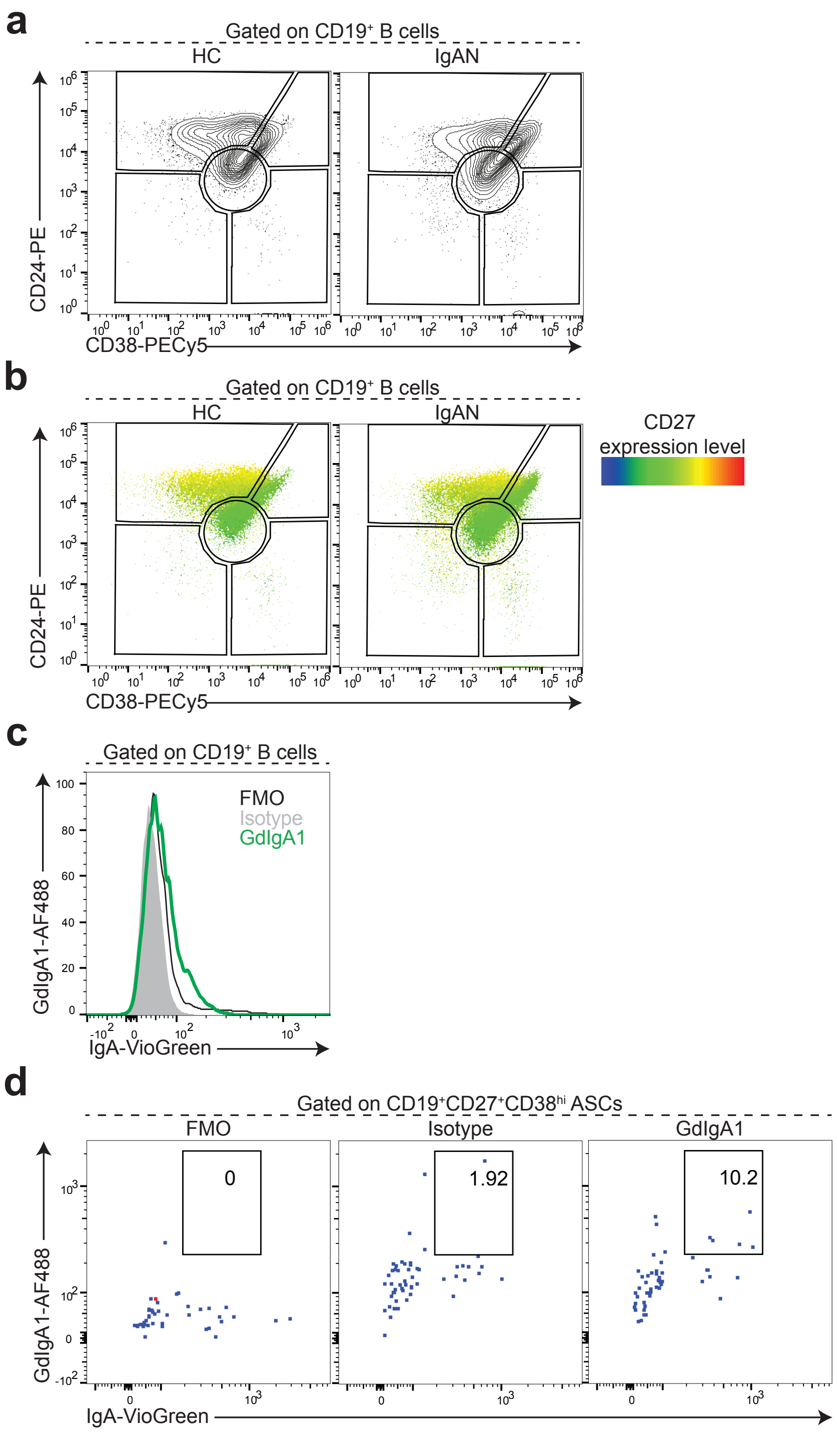

Suppl. Figure 1. CD24/CD38 and GdIgA1 gating strategy. Representative flow cytometry plots demonstrating the distribution of transitional ($CD24^{hi}CD38^{hi}$), mature ($CD24^{int}CD38^{int}$), memory ($CD24^{hi}CD38^{lo}$), and activated B cells ($CD24^{lo}CD38^{lo}$) and pre-plasmablasts ($CD24^{lo}CD38^{hi}$) as contour (a), and as CD27 heatmap statistic (b) plots in healthy controls and patients with IgAN, (c,d) gating strategy for GdIgA1 in B cells (c) and plasmablasts (d).
