# Supplementary material for: IgA class-switched CD27−CD21+ B cells in IgA nephropathy
Source: Nephrol Dial Transplant. 2024 Jul 17;40(3):505–15. doi: 10.1093/ndt/gfae173 (PMC11879059; doi:10.1093/ndt/gfae173)
Supplement: gfae173_Supplemental_Files [file gfae173_supplemental_files.zip › Suppl. Table 1.docx]

| **Antigen** | **Fluorochrome** | **Clone** | **Cat. no.** | **Supplier** | **Dilution** |
| --- | --- | --- | --- | --- | --- |
| CD3 | APC-Cy7 | HIT3a | 300318 | BioLegend | 1:20 |
| CD19 | AF700 | HIB19 | 557921 | BD Biosciences | 1:50 |
| CD21 | FITC | Bu32 | 354910 | BioLegend | 1:20 |
| CD24 | PE | ML5 | 311106 | BioLegend | 1:50 |
| CD27 | PE-Dazzle 594 | M-T271 | 356422 | BioLegend | 1:50 |
| CD38 | PE-Cy5 | HIT2 | 303508 | BioLegend | 1:20 |
| IgA | APC | IS11-8E10 | 130-113-472 | Miltenyi Biotec | 1:50 |
| IgA | VioGreen | IS11-8E10 | 130-113-481 | Miltenyi Biotec | 1:50 |
| IgD | FITC | IA6-2 | 555778 | BD Biosciences | 1:50 |
| IgD | PECy7 | IA6-2 | 348210 | BioLegend | 1:20 |
| IgG | VioBlue | IS11-3B2.2.3 | 130-119-881 | Miltenyi Biotec | 1:50 |
| IgM | APC | PJ2-22H3 | 130-122-915 | Miltenyi Biotec | 1:50 |
| Ki-67 | PE-Cy7 | 20Raj1 | 25-5699-42 | eBioscience | 1:20 |
| T-bet | PE | 4B10 | 12-5825-82 | eBioscience | 1:20 |

**Suppl. Table 1.** Antibodies used in flow cytometry.
