# Supplementary material for: IgA class-switched CD27−CD21+ B cells in IgA nephropathy
Source: Nephrol Dial Transplant. 2024 Jul 17;40(3):505–15. doi: 10.1093/ndt/gfae173 (PMC11879059; doi:10.1093/ndt/gfae173)
Supplement: gfae173_Supplemental_Files [file gfae173_supplemental_files.zip › Suppl. Table 2.docx]

**Suppl. Table 2.** Relationships between LPS and B cell subsets.

| **B cells** | **r** | **p value** |
| --- | --- | --- |
| CD27^+^CD38^++^IgA^+^CD27^+^ | 0.201 | 0.162 |
| CD24^lo^CD38^lo^ | 0.34 | 0.016 |
| IgA^+^CD27^+^ | 0.201 | 0.163 |
| CD24^int^CD38^int^ | 0.185 | 0.291 |
| CD24^hi^CD38^lo^ | -0.213 | 0.137 |
| IgD^+^ mem | 0.258 | 0.07 |
| IgD^-^ mem | -0.263 | 0.65 |
| Transitional CD24^hi^CD38^hi^ | -0.115 | 0.426 |
